# The contribution of demographic and morbidity factors to self-reported visit frequency of patients: a cross-sectional study of general practice patients in Australia

**DOI:** 10.1186/1471-2296-5-17

**Published:** 2004-08-20

**Authors:** Stephanie A Knox, Helena Britt

**Affiliations:** 1 AIHW General Practice Statistics and Classification Unit, Family Medicine Research Centre, University of Sydney, Sydney, Australia

## Abstract

**Background:**

Understanding the factors that affect patients' utilisation of health services is important for health service provision and effective patient management. This study aimed to investigate the specific morbidity and demographic factors related to the frequency with which general practice patients visit a general practitioner/family physician (GP) in Australia.

**Methods:**

A sub-study was undertaken as part of an ongoing national study of general practice activity in Australia. A cluster sample of 10,755 general practice patients were surveyed through a random sample of 379 general practitioners. The patient reported the number of times he/she had visited a general practitioner in the previous twelve months. The GP recorded all the patient's major health problems, including those managed at the current consultation.

**Results:**

Patients reported an average of 8.8 visits to a general practitioner per year. After adjusting for other patient demographics and number of health problems, concession health care card holders made on average 2.6 more visits per year to a general practitioner than did non-card holders (p < .001). After adjustment, patients from remote/very remote locations made 2.3 fewer visits per year than patients from locations where services were highly accessible (p < .001). After adjustment for patient demographics, patients with diagnosed anxiety made on average 2.7 more visits per year (p = 0.003), those with diagnosed depression 2.2 more visits than average (p < .0001), and those with back problems 2.4 more visits (p = 0.009) than patients without the respective disorders.

**Conclusions:**

Anxiety, back pain and depression are associated with greater patient demand for general practice services than other health problems. The effect of sociodemographic factors on patient utilisation of general practice services is complex. Equity of access to general practice services remains an issue for patients from remote areas, while concession health care card holders are attending general practice more frequently than other patients relative to their number of health problems.

## Background

The frequency of patient visits to general/family practice is affected by a range of factors including patient characteristics, physician/practice factors and broader issues such as access to services. A major contributor to the variance in visit rates to general/family practitioners is the complexity of health problems experienced by the patient. Those patients who attend general practice frequently report poorer health than those who attend less frequently [1,2]. Not all health problems however, have an equal effect on patient visit rates to general practice.

Numerous studies have found that for a subset of patients, the presence of psychological health problems is associated with frequent attendance at medical services [3-6]. Higher depression scores and increased levels of health anxiety have been found among patients who attend general practice frequently [3], and practitioners are more often involved in managing psychosocial issues for frequent attenders than other patients [1]. Frequent attendance at general practice has also been found to be associated with an increased use of other health services such as out of hours services and "inappropriate attendance" at accident and emergency wards [6-8].

The general practitioner (GP) also influences patients' attendance rates through doctor-initiated visits and some practitioners attract a larger proportion of frequent attenders than others [9]. Remuneration may also affect GP behaviour. For example, GPs paid a flat rate capitation per patient may be more motivated to manage their own workload by reducing patient return visits [10].

Positive aspects to increasing general practice attendance includes recalling patients for chronic conditions as part of a program of structured care [11,12]. Patients who attend the GP frequently often receive improved continuity of care [9], while those who attend general practice infrequently may be receiving less than optimal care, especially where geographical barriers to access result in lower utilisation of general practice services [13,14].

The available evidence on visit frequency to general/family practice comes from a range of settings in several countries. There are differences in general practice between countries that may affect patterns of patient visits and some studies are based on a limited number of practices, which may affect the generalisability of the findings [3,15]. Although the association between frequent medical visits and increased psychological health problems has been well-researched, the effect of other specific chronic conditions on general practice visit rates is less clear. In Australia there are few studies that examine the relationship between patient morbidity, social and demographic factors and the frequency of visits to general practice. We therefore investigated patient sociodemographic and morbidity factors associated with patient self-reported visit frequency among general practice patients in Australia, as part of an ongoing national study of general practice activity.

## Methods

The study reported here is part of the Bettering the Evaluation and Care of Health (BEACH) program, a national study of general practice activity in Australia. The BEACH method has been described in detail elsewhere [16]. In summary, BEACH is a continuous cross-sectional study, which commenced in April 1998. A random sample of approximately 1,000 GPs is recruited throughout each year in a rolling sample. The GPs are selected from a sampling frame of all Australian GPs who claimed 375 or more general practice (A1) items from the Medicare Benefits Schedule in the previous quarter. Each GP provides details for 100 consecutive patient encounters.

### Design

This paper is based on a sub-sample of GPs who participated in BEACH between August 2001 and March 2002. For a subset of 30 out of the 100 encounters, the GP recorded, in addition to the problems managed at the encounter, any other major health problems of the patient that had not been managed at the current encounter. GPs were instructed to include chronic illnesses requiring ongoing care, past problems that affect present and future care and social problems that influence health. As many as 12 extra problems could be recorded per patient and when added to the maximum of four problems managed at the encounter this allowed up to 16 health problems to be recorded per patient. The GP recorded problems in free text, which were secondarily classified according to the International Classification of Primary Care (ICPC-2) using an extended vocabulary of terms [17,18]. ICPC-2 includes components for diagnoses and symptoms, and process codes (e.g. "Test results", "Prescription renewal"). Process codes were excluded from counts of health problems, unless they clearly identified the nature of the underlying condition. Synonymous or related problems were grouped according to standard ICPC-2 categories to ensure that problem categories included all patients with the disorder [16].

### Morbidity variables

Mental health (particularly depression), asthma, diabetes, osteoarthritis and cardiovascular disease are five National Health Priority Areas that are major contributors to mortality, morbidity and health service costs in Australia [19]. They are also chronic health problems commonly managed in general practice [20]. In addition hypertension, back complaint and oesophageal disease are among the most common problems managed in general practice [20]. We therefore focussed on these health areas of particular relevance to general practice when investigating the association between morbidity and frequency of patient visits to a GP.

### Number of annual visits to general practice

The GP asked each patient in the sub-sample to recall how many times he/she had visited a GP in the previous 12 months.

### Demographic variables

Commonwealth concession health care cards are available to people on limited incomes and entitle the holder to health services at greatly reduced cost. In 1998, around one third of Australians aged 15 years and over held a concession health care card [21]. Concession health care card status was included in the analysis as a marker of socioeconomic status [22].

The Remote Index of Australia (ARIA) was used to classify patients according to geographical remoteness and accessibility of services [23]. Patients were classified by patient residential postcode into three broad categories: those from areas that were "highly accessible" to services, those from areas that were "accessible/moderately accessible" and those from areas "remote/very remote" from services. These three categories were used to investigate the association between geographical barriers to access and the frequency of patient visits to a GP.

### Statistical analysis

The patient sample was a single stage cluster design with the GP as the primary sampling unit. Observations recorded by the same GP were therefore not independent and the statistical analysis adjusted for the correlation between patients within each cluster. We used procedures in SAS software V8.2 that adjust the standard error for the intra-cluster correlation [24] and all reported p-values and confidence intervals in both the descriptive and multivariable analyses include this adjustment.

### Multiple regression

Number of annual visits to a GP was the main outcome of interest. After checking the linear relationship between number of visits and other ordinal variables (age and number of health problems), multiple regression was performed in two stages to identify predictors of visit frequency.

1) Demographic predictors

We performed multiple regression, with self-reported annual GP visits as an ordinal outcome to identify the independent demographic predictors of the frequency of visits to a GP after controlling for morbidity. We fitted the morbidity covariate as the total number of major health problems recorded for the patient.

2) Morbidity predictors

We fitted separate regression models to estimate the effect of specific morbidity on visit frequency, after adjusting for patient age, sex, other significant demographic predictors and the number of other health problems of the patient. The morbidities of interest were chronic problems most commonly managed in general practice, specifically depression, anxiety, back problems, diabetes, hypertension, asthma, ischaemic heart disease, and osteoarthritis [16]. We interpreted the partial regression co-efficient of morbidity in each model as the mean difference in annual GP visits between patients who had the problem versus those who did not, when other factors were kept constant. A large number of models was fitted, therefore an alpha of 0.01 was used as the test of significance to reduce type I error.

## Results

Three hundred and seventy nine GPs participated in the sub-study. Six hundred and eighteen patients (5.4%) did not answer the question on visit frequency, giving a final sample of 10,755 respondents. Patient respondents and non-respondents were not significantly different in mean age, sex distribution or mean number of health problems recorded.

While all states of Australia were well represented in the GP sample, the sample had a smaller proportion of GPs aged less than 35 years and was somewhat more urban than the population of Australian GPs (Table 1).

**Table 1:** Comparison of general practitioner (GP) characteristics for the sample and the population of Australian general practitioners in 2001.

	**GP sample**	**Australian population of GPs**^(a)^
N	379	17,534
Male	61.4%	67.5%
Age group		
<35 years	8.2%	12.1%
35–44	29.3%	27.1%
45–54	36.7%	32.0%
55 +	25.9%	28.8%
Urban/Metropolitan practice	76.0%	72.6%
Solo practice	16.1%	Not available
5 plus GPs in practice	45.2%	Not available

^(a) ^defined as practitioners who claimed the equivalent of 1,500 general practice (A1) Medicare items of service in 2001 (Medicare Benefits Schedule unpublished data, Australian Department of Health and Ageing)

Patients aged less than 25 years were under-represented in the sample relative to all patients who had visited a GP in Australia at least once in 2001, while patients aged 65 years and older were over-represented (Table 2).

**Table 2:** Characteristics of the patient sample versus the general practice patient population in Australia in 2001.

	**Sample n (%)**	**Australian population of general practice patients^(a) ^%**
Female patient	6,404 (59.5)	53.4
Age group		
<15 years	1,360 (12.7)	19.9
15–24	1,005 (9.4)	13.1
25–44	2,875 (26.9)	29.7
45–64	2,677 (25.0)	23.8
65–74	1,274 (11.9)	7.6
75+	1,514 (14.1)	5.9
Address in "highly accessible" location	8,702 (83.3)	Not available
Speaks language other than English at home.	890 (8.3)	Not available
Holds concession health care card	4,448 (41.4)	Not available

^(a) ^Defined as persons who claimed at least one general practice (A1) Medicare item in 2001 (Medicare Benefits Schedule unpublished data, Australian Department of Health and Ageing)

The majority of patients in the sample (83.3%) were from locations where services were "highly accessible", and 8.3% spoke a language other than English at home.

The mean patient self-reported visit rate for the sample was 8.8 (95% CI: 8.3–9.2) visits to a GP per year. On average the GPs recorded 2.9 morbidities per patient, and half (51.1%) of the recorded problems were being managed by the GP at the current consultation (results not tabled).

There was no difference in self-reported visit frequency between the sexes (p = 0.11) (results not tabled). The mean visit rate for children aged less than 5 was 5.8 visits per year, which fell to 4.3 for children 10–14 years of age, then increased linearly after age 15 years (p < .0001). There was a simple linear increase in frequency of visits with increasing number of recorded health problems (p < .0001). Therefore age was fitted as a categorical variable and number of problems as a numeric variable in the multiple regression models.

After adjusting for age and sex, there was no difference in visit frequency according to language spoken at home (Table 3). After adjusting for age, sex, remoteness and total number of health problems, concession health care card holders made on average 2.6 more visits per year to a GP than non-card holders (p < .001, Table 4). After adjusting for age, sex, concession health care card status and total number of health problems, patients from remote/very remote locations made on average 2.3 fewer visits per year to a GP than patients from locations where services were highly accessible (p < .001).

**Table 3:** Mean number of recorded problems (crude rate) and differences in mean annual visits to a GP (crude rates and adjusted for age and sex) by patient demographics

		**Annual visits**		
		
		**Crude**	**Adjusted**	
			
**Patient characteristics (n)**	**Mean problems**	**Mean visits**	**Mean visits**	**p-value**
Highly accessible (8,702)	2.9	9.1	9.0	
Moderately accessible (1,599)	2.7	7.7	7.9	0.05
Remote/very remote (145)	2.5	5.8	6.5	<.0001
Concession health care card holder (4,448)	3.5	11.4	10.7	<.0001
Non health care card holder (6,307)	2.4	6.8	7.5	
Speaks language other than English at home (890)	2.8	9.0	9.1	0.369
Speaks English at home (9,865)	2.9	8.7	8.8	
Male (4,285)	2.7	8.5	N/A	N/A
Female (6,404)	2.9	8.9	N/A	

**Table 4:** Multiple regression model of sociodemographic predictors of visit rates and number of recorded health problems

	**Partial coefficient^(a) ^(95%CI)**	**p-value**
Patient sex (ref: male)	0.0 (-0.6;0.5)	0.88
Patient age (ref: <1 year)		
1–4 years	2.2 (1.5;2.9)	<.001
5–14	-0.2 (-0.8;0.4)	0.46
15–24	0.5 (-0.1;1.2)	0.08
25–44	2.2 (1.5;3.0)	<.001
45–64	1.9 (1.1;2.8)	<.001
65–74	2.0 (0.9;3.1)	<.001
75+	2.7 (1.4;4.1)	<.001
Accessibility (ref: Highly accessible)		
Moderately accessible	-1.0 (-2.1;0.2)	0.12
Remote/very remote	-2.3 (-3.1;-1.5)	<.001
Concession health care card holder	2.6 (1.8;3.3)	<.001
Each extra health problem	1.6 (1.3;1.8)	<.001

^(a) ^Interpreted as the change in mean number of annual visits after adjusting for all other variables in the model.

### Morbidity and visit rates

The mean annual visit rates for patients with common chronic disorders were well above average (Table 5). After adjusting for demographic predictors and total number of health problems, patients with anxiety made on average 2.7 more visits to a GP per year (p = 0.003), those with depression 2.2 more visits (p < .0001) and those with back problems 2.4 more visits (p = 0.009). Ischaemic heart disease, diabetes, asthma, oesophageal disease, osteoarthritis or hypertension did not affect patient visit rate beyond that expected from the patients' demographics and overall number of health problems.

**Table 5:** The effect of specific morbidity on annual visits to a GP (Crude rates, rates adjusted for age, sex, concession health care card, remoteness and number of other problems)

	**Crude rates**		**Effect on visit rate (adjusted)**	
**Health problem^(a) ^(n)**	**Mean annual visits**	**No. other problems**	**Morbidity coefficient^(b) ^(95%CI)**	**p-value**

Anxiety (381)	14.1	3.4	2.7 (0.9;4.5)	0.003^†^
Depression (1,100)	13.1	3.2	2.2 (1.4;3.1)	<.0001^†^
Back complaint (578)	12.8	2.9	2.4 (0.6;4.2)	0.009^†^
Diabetes (706)	13.7	3.8	0.9 (-0.1;1.9)	0.08
Ischaemic heart disease (585)	14.3	4.3	0.6 (-0.7;1.9)	0.38
Asthma (876)	10.5	2.8	0.3 (-0.4;1.0)	0.40
Oesophageal disease (636)	12.9	4.1	-0.2 (-1.2;0.8)	0.65
Osteoarthritis (859)	13.0	4.1	-0.4 (-1.3;0.5)	0.39
Hypertension (2,094)	11.8	3.5	-0.4 (-1.2;0.4)	0.30

^(a)^Each line represents a separate regression model (adjusted for age, sex, concession health care card holder, remoteness and number of problems).

^(b) ^Interpreted as the mean difference in number of annual visits between patients with the specific problem versus those without after adjusting for age, sex, concession health care card holder, remoteness and overall number of health problems.

† significant p < .01

## Discussion

As would be expected the number of health problems recorded for the patient was a major predictor of frequency of attendance at general practice. However this study demonstrated that specific chronic disorders had differential effects on visit frequency. Anxiety, back complaints, and depression had the greatest effects on increasing patient annual visits to a GP.

The relationship between psychological health problems and increased medical visits has been demonstrated across many settings [1-3,5]. The current study further demonstrates the generalisability of this relationship. However the interpretation of this finding in the Australian context is less clear. Patients with depression may be visiting GPs more frequently because GPs fail to recognise and treat the root causes of the patient's poor mental health [5]. Alternatively, above average visits for depression and anxiety may be appropriate if the practitioner has initiated the return visit for ongoing management. GPs in Australia have been targeted to improve the detection and management of depression among general practice patients [25], so higher visit rates for some patients with depression could reflect a more active intervention strategy by their GPs.

Other research has reported a link between unexplained back pain and frequent attendance at medical services [26]. In the current study back complaints included problems described as symptomatic back pain as well as diagnosed disc and nerve problems [18,20]. It is unclear the degree to which the frequent visits of patients with back complaints may be due to somatisation [27] and how much is explained by the need to manage acute/intractable pain from organic causes.

Other research indicates that patients with diabetes make more general practice visits than average [28] and structured care programs might be expected to result in above average visit frequency for patients with diabetes [12,29]. The current analysis controlled for other health and demographic factors and found that the higher visit rates of patients with diabetes was more related to the high number of health problems reported by the patient rather than to extra visits related to diabetes per se. Regression models revealed similar explanations for the visit rates of patients with asthma, ischaemic heart disease, osteoarthritis, oesophageal disease and hypertension: visit rates were mostly explained by the overall level of health problems experienced by the patient rather than to any increased demand for health care related to the specific morbidity.

As expected, patients from remote areas reported visiting general practice less frequently relative to their health needs, confirming that geographic access to services remains a significant barrier to meeting the health care needs of patients from remote locations [13,14].

Concession health care card holders have more chronic and psychological health problems managed in general practice relative to the rest of the Australian general practice population [22]. In this study concession health care card holders reported a higher than average number of visits even after accounting for their number of health problems. This relatively high visit rate coupled with high levels of ill-health places concession health care card holders among the most frequent attenders at general practice. Shorter consultation length is associated with lower socioeconomic status in Australia [30]. Perhaps concession health care card holders are partly compensating for less time spent with the GP at each visit by visiting more often.

This study had the advantage of randomly sampling clusters of patients across a wide range of practices in all states and regions of Australia. A further advantage was having a qualified medical practitioner to record patient morbidity and the use of the medical record to help validate patient recall of GP visits.

The methodology of this study was limited by using patient self-report of visit frequency and by sampling patients at the point of consultation. The average number of GP visits among Australians who visit a general practitioner at least once in a 12 month period is known to be six visits per year, increasing with increasing age (Australian Health Insurance Commission, unpublished data), while the self-reported visit rate in the current study was 8.8 visits per year. Because the patients were sampled at the consultation, older patients who attend general practice more frequently had a greater chance of being sampled and this was reflected in the older age distribution of the patient sample. Even allowing for this selection bias, the mean annual self-reported visits was somewhat higher than expected given the sample's age structure, indicating some overestimation by patients in their recall of the number of GP visits. One Australian study that validated patient self-report against actual Medicare benefit claims found that patients who overestimate recent use of medical services are "telescoping" real but less recent events into the nominated time period and are in fact higher users of health services over the long term [31]. In the current study the number of reported visits increased with age and number of morbidities as expected. Therefore even if somewhat overestimated, self-reported visit frequency had sufficient face validity to allow a comparative analysis of the effect of morbidity and demographic factors on the relative frequency of visits to a GP.

## Conclusion

This study has demonstrated that number of visits to a GP by patients in Australia is largely explained by the number of health problems the patient is experiencing. However psychological health problems and sociodemographic factors have differential effects on visit rates. GPs need to be alert to the particular psychological and social needs of patients who are attending frequently with multiple health problems. Equity of access, in terms of equal utilisation of services, continues to be an issue for patients living in remote locations.

## Competing interests

None declared.

## Authors contributions

SK designed and performed the statistical analysis, researched and wrote the main draft. HB directed the study, participated in the design and assisted in writing and reviewing the final article.

## Pre-publication history

The pre-publication history for this paper can be accessed here:


